# Comparison of four DNA extraction and three preservation protocols for the molecular detection and quantification of soil-transmitted helminths in stool

**DOI:** 10.1371/journal.pntd.0007778

**Published:** 2019-10-28

**Authors:** Mio Ayana, Piet Cools, Zeleke Mekonnen, Abdissa Biruksew, Daniel Dana, Nour Rashwan, Roger Prichard, Johnny Vlaminck, Jaco J. Verweij, Bruno Levecke

**Affiliations:** 1 School of Medical Laboratory Sciences, Institute of Health, Jimma University, Jimma, Ethiopia; 2 Department of Virology, Parasitology and Immunology, Faculty of Veterinary Sciences, Ghent University, Ghent, Belgium; 3 Institute of Parasitology, McGill University, Montreal, Quebec, Canada; 4 Laboratory for Medical Microbiology and Immunology, Elisabeth-TweeSteden Hospital, Tilburg, The Netherlands; University of Cambridge, UNITED KINGDOM

## Abstract

**Background:**

A DNA extraction and preservation protocol that yields sufficient and qualitative DNA is pivotal for the success of any nucleic acid amplification test (NAAT), but it still poses a challenge for soil-transmitted helminths (STHs), including *Ascaris lumbricoides*, *Trichuris trichiura* and the two hookworms (*Necator americanus* and *Ancylostoma duodenale*). In the present study, we assessed the impact of different DNA extraction and preservativation protocols on STH-specific DNA amplification from stool.

**Methodology and principal findings:**

In a first experiment, DNA was extracted from 37 stool samples with variable egg counts for *T*. *trichiura* and *N*. *americanus* applying two commercial kits, both with and without a prior bead beating step. The DNA concentration of *T*. *trichiura* and *N*. *americanus* was estimated by means of qPCR. The results showed clear differences in DNA concentration across both DNA extraction kits, which varied across both STHs. They also indicated that adding a bead beating step substantially improved DNA recovery, particularly when the FECs were high. In a second experiment, 20 stool samples with variable egg counts for *A*. *lumbricoides*, *T*. *trichiura* and *N*. *americanus* were preserved in either 96% ethanol, 5% potassium dichromate or RNA*later* and were stored at 4°C for 65, 245 and 425 days. DNA was extracted using the DNeasy Blood & Tissue kit with a bead beating step. Stool samples preserved in ethanol proved to yield higher DNA concentrations as FEC increased, although stool samples appeared to be stable over time in all preservatives.

**Conclusions:**

The choice of DNA extraction kit significantly affects the outcome of NAATs. Given the clear benefit of bead beating and our validation of ethanol for (long-term) preservation, we recommend that these aspects of the protocol should be adopted by any stool sampling and DNA extraction protocol for downstream NAAT-based detection and quantification of STHs.

## Author summary

DNA-based tools are increasingly being used for the diagnosis of intestinal worm infections in both clinical and research laboratories. However, recovering DNA from intestinal worm eggs in stool remains a challenge since this DNA is protected by a very rigid egg shell. Furthermore, stool contains inhibitors that can affect test results and these should be removed during DNA extraction. Prior to DNA extraction, samples are often preserved, but the impact of the type of preservatives and the duration of preservation remains poorly studied. In the present study, we assessed the impact of four DNA extraction and three preservation protocols on the downstream performance of a DNA-based diagnostic tool for intestinal worms. We found significant differences in DNA recovery across the DNA and preservation protocols, but DNA from worm eggs in stool proved to be stable over time in all preservatives.

## Introduction

Soil-transmitted helminth (STH) infections are among the most common parasitic infections globally and affect more than a quarter of the world’s population, mainly poor populations in (sub)tropical regions. These infections are caused by intestinal helminths (worms) which excrete eggs through human stool, that contaminate soil in areas where sanitation is poor and in turn infect the human host orally or through skin contact. The main STH species are the giant roundworm (*Ascaris lumbricoides*), the whipworm (*Trichuris trichiura*), and the two hookworms (*Necator americanus* and *Ancylostoma duodenale*). Today, STHs are responsible for nearly two million disability-adjusted life years [1, 2].

To control the burden caused by these worms, the World Health Organization (WHO) recommends preventive chemotherapy (PC) programs, during which anthelmintic drugs are administered to at-risk populations (i.e., pre-school-aged children, school-aged children and women of reproductive age). WHO recommends a bi-annual PC when the prevalence of any STHs exceeds 50% and an annual PC when the prevalence is between 20% and 50%. For a prevalence below 20%, it is not recommended to initiate a PC program [3].

Diagnostic tools play a pivotal role in these PC programs as they provide information on the population prevalence and infection intensity distribution that ultimately guides PC program decisions. Today, microscopy of Kato-Katz thick smears is the most widely used tool for the detection and enumeration of STH eggs in stool [3, 4].

More recently, nucleic-acid amplification tests (NAATs) are being used increasingly in clinical [5] and research settings [6–10]. Although these NAATs are associated with a considerable cost for both pre-analytical procedures (DNA extraction), equipment, reagents and training, they have some important advantages which make them an attractive diagnostic tool for STH PC programs. First, NAATs are much more sensitive, which makes them more appropriate to monitor the progress of PC programs when both infection intensity and prevalence of STHs declined after multiple rounds of PC [11]. Second, next to detecting STH DNA, they allow for the simultaneous detection of a variety of other co-endemic pathogens such as *Strongyloides stercoralis*, another important STH, for which large-scale epidemiological data is lacking due to the poor diagnostic performance of the Kato-Katz method for this helminth [12–14]. Third, NAATs allow differentiation of STH species that cannot be distinguished using microscopy, such as the hookworms. This is important because the human hookworms *N*. *americanus* and *A*. *duodenale* have a clear different impact on health [15]. Furthermore, there is an increasing number of studies that indicate that animal STHs can infect humans. Important zoonotic species that are known to cause patent infections in humans are *Ascaris suum* and *Trichuris suis* from pigs [16] and *Ancylostoma ceylanicum* [17–18], *Ancylostoma caninum* [19] and *Trichuris vulpis* from dogs [20]. Finally, NAATs also allow for the detection of mutations in genes that have been associated with anthelmintic resistance [21, 22]. Despite these advantages, the diagnostic performance of NAATs is highly dependent on an effective DNA extraction. This is a particular challenge for STH eggs in stool, as the shell of the STH eggs are hard to lyse [10,23]. Moreover, stool is a complex matrix containing numerous compounds that may inhibit the amplification of nucleic-acids [24]. Moreover, when NAATs are to be applied in STH PC programs, samples will be collected usually from remote rural areas and transported to a centralized laboratory for analysis, necessitating stool preservation [25]. In the past a variety of common (ethanol and potassium dichromate) and commercial preservatives (e.g., RNA*later* and PAXgene) have been used [9,26–28], but the impact of these preservatives and the duration of preservation on DNA recovery remains poorly studied. In this study, we compared the performance of four DNA extraction protocols (DNA extraction experiment), three preservatives and three storage periods (preservation experiment) for the downstream quantitative PCR (qPCR) based detection and quantification of STHs in human clinical stool samples.

## Methods

### Ethical statements

For the two experiments (the DNA extraction and the preservation experiment), stool samples were collected from primary school children. The protocols for the two experiments were separately approved by the Institutional Review Board of Jimma University, Ethiopia (DNA extraction experiment: reference RPGC/478/2014; preservation experiment: reference RPGC/547/2016). The school administrators, teachers, parents/legal guardians and children were informed about the objectives of the study. Children that appeared in overall healthy condition, whose parents or legal guardians signed an informed consent and who volunteered to provide a sufficient amount of stool sample were included. Children who were found excreting any of the STH eggs were treated with a single oral dose of 400 mg albendazole (GlaxoSmithKline).

### DNA extraction experiment

#### Selection of samples

Based upon previous STH prevalence data from Jimma Town (Ethiopia) [29], three primary schools were strategically chosen for stool sample collection, with the ultimate aim to enroll STH positive subjects excreting different levels of eggs concentration in stool. A total of 195 stool samples from children 5–14 years of age were microscopically screened using the McMaster egg counting method at the Neglected Tropical Disease Laboratory of Jimma University (Ethiopia) using a previously described protocol [30,31]. Briefly, 2 grams of stool was suspended in 30 ml of flotation solution (saturated sodium chloride; specific density = 1.2). The suspension was sieved using a plastic tea strainer to withhold the large debris. This sieved stool suspension was then mixed by pouring 10 times from one cup to the other. Finally, the suspension was transferred to both chambers of a McMaster slide using a Pasteur pipette. STH eggs were allowed to float for 2 min and they were subsequently microscopically counted in both chambers (= 2 x 0.15 ml) using a 100x magnification. To obtain the fecal egg counts (FECs) expressed as number of eggs per gram of stool (EPG), the number of eggs for each STH was multiplied by 50. Based on the FECs, the intensity of infection was classified as low (*T*. *trichiura*: FEC <1,000 EPG; hookworm: FEC <2,000) and as moderate-to-heavy (*T*. *trichiura*: FEC ≥1,000 EPG; hookworm: FEC ≥2,000 EPG) [32]. We aimed at including a minimum of 10 stool samples of each level of infection intensity for both *T*. *trichiura* and hookworms. We restricted the analysis of these two STHs, as their eggs are the most difficult (*T*. *trichiura*) and the easiest to lyse (hookworm). In addition, we also included 10 negative samples. Finally, 3 grams of the selected stool samples were preserved in 96% ethanol to make a total volume of 10 ml and stored at room temperature until DNA extraction. All samples were preserved within 6 hours after the stool collection.

#### DNA extraction protocols

We compared two commercially available DNA extraction kits, i.e. the QIAamp DNA Stool Mini kit and DNeasy Blood & Tissue kit (both Qiagen, Germany). The Qiagen DNeasy Blood & Tissue kit was chosen because it was our in-house DNA extraction protocol, while the QIAamp DNA Stool Mini kit was selected because it is Qiagen’s recommended kit for stool samples. Both kits were tested with and without a preceding bead beating step. This step aims to mechanically rupture the egg shells [32], but this is not included in both commercial Qiagen protocols. For each of the four extraction protocols, an aliquot of approximately 666 μl of the 96% ethanol preserved stool sample was transferred into an Eppendorf tube. This volume corresponds with the 0.2 g of stool recommended by the manufacturer of the QIAamp DNA Stool Mini kit. As there is no recommended amount of stool mentioned in the DNeasy Blood & Tissue kit manual, we used the same amount of stool as for the QIAamp DNA Stool Mini kit protocols. To avoid any systematical error (e.g., the first aliquot systematically being assigned to the same DNA extraction protocol), we randomized the aliquots across the four DNA extraction protocols. Subsequently, the ethanol was removed from all stool aliquots. To this end, all aliquots were centrifuged at 10,000 rpm (8,944 g) for 1 min (Heraeus^™^ Pico^™^ 17 Microcentrifuge, Thermo scientific, Germany), after which the supernatant was discarded by pipetting. The remaining pellet was further washed by adding 1 ml of phosphate buffer saline (PBS), after which it was mixed by vortexing and centrifuged at 10,000 rpm (8,944 g) for 1 min. Subsequently, the supernatant was discarded. The pellet was then resuspended in 200 μl of PBS. To enhance egg shell rupture and minimize inhibition, a freeze-thaw-boiling step was included in all extraction protocols (not included in the manufacturer’s protocols). This step consisted of a freeze phase at -80°C for 30 min followed by a thaw-boiling phase in a preheated shaking heating block at 100°C for 10 min. For the two bead beating extraction protocols, the samples were transferred to Green Bead tubes (Roche, Germany) and subjected to bead beating by manual vortexing (VM-300, Jemmy Industrial Corp., Taiwan) at maximum speed (3,150 rpm) for 5 min.

Thereafter, we further followed the manufacturer’s protocol for all four extraction protocols. Briefly, for both QIAamp DNA Stool Mini kit extraction protocols (with and without preceding bead beating), 2 ml of buffer ASL was added to each sample tube and thoroughly mixed by vortexing. Subsequently, 1.6 ml of this suspension was transferred to a 2 ml Eppendorf tube and heated at 70°C for 5 min. After centrifugation at 10,000 rpm (8,944 g) for 2 min, 1.2 ml of supernatant was transferred to a new 2 ml Eppendorf tube containing 1 InhibitEX tablet, which adsorbs inhibitors from the suspension. After centrifugation at 10,000 rpm (8,944 g) for 3 min, 200 μl of supernatant was added to an Eppendorf tube, to which 15 μl of Proteinase K and 200 μl AL lysis buffer was added. Tubes were then incubated at 70°C for 10 min. The complete lysate was added to QIAamp spin column and centrifuged until the lysate completely passed through the spin column membrane enabling nucleic acids to attach to the column membrane. Subsequently, the column was washed with buffers AW1 and AW2. Finally, bound DNA was eluted in 200 μl of AE buffer.

For both DNeasy Blood & Tissue kit extraction protocols (with and without preceding bead beating), 180 μl of buffer ATL containing 20 μl of Proteinase K was added to the sample and incubated at 55°C for 2 h. Subsequently, 400 μl of buffer AL was added and samples where incubated at 70°C for 10 min. Then, the suspension was centrifuged at 10,000 rpm (8,944 g) for 30 seconds, and the supernatant was transferred to an Eppendorf containing 400 μl of 96% ethanol. A total of 600 μl of the mixture was pipetted to a spin column supported by a 2 ml collecting tube and centrifuged at 10,000 rpm (8,944 g) until the lysate completely passed through spin column and the same step applied for rest of the lysate. Subsequently, the column was washed with buffers AW1 and AW2. Finally, bounded DNA was eluted in 200 μl of buffer AE. All DNA extracts were stored at -20°C until shipment on dry ice to the Laboratory for Medical Microbiology and Immunology, Elisabeth-TweeSteden Hospital, Tilburg (The Netherlands) for qPCR analysis.

#### qPCR

DNA of *T*. *trichiura* and hookworms (*N*. *americanus* and *A*. *duodenale*) was detected using qPCR assays that are routinely used at the Laboratory for Medical Microbiology and Immunology, Elisabeth-TweeSteden Hospital, Tilburg (The Netherlands). The primers and probes used in this qPCR analysis are described in Table 1. The assays were performed on a RotorGene amplification platform using the following cycling conditions: initial denaturation of 15 min at 95°C, followed by 45 cycles of 10 s at 95°C, 15 s at 60°C and 15 s at 72°C. The qPCR results were expressed as the number of genome equivalents per ml of DNA extract (GE/ml) as described by Cools and co-workers [11]. A detailed standard operating procedure used to assess the linkage between Ct and GE/ml is provided in **S1 File.**

**Table 1:** Primers and probes used for the qPCR assays.

Species	Primer/ probe	Sequence (5’-3’)	Target region	References
***Ascaris lumbricoides***	Fwd	GTAATAGCAGTCGGCGGTTTCTT	ITS-1	[34]
	Rev	GCCCAACATGCCACCTATTC		[34]
	Probe	Texas Red-TTGGCGGACAATTGCATGCGAT-BHQ2		[35]
***Trichuris trichiura***	Fwd	TTGAAACGACTTGCTCATCAACTT	18S	[36]
	Rev	CTGATTCTCCGTTAACCGTTGTC		[36]
	Probe	Yakima Yellow-CGATGGTACGCTACGTGCTTACCATGG-BHQ1		[36]
***Ancylostoma duodenale***	Fwd	GAATGACAGCAAACTCGTTGTTG	ITS-2	[37]
	Rev	ATACTAGCCACTGCCGAAACGT		[37]
	Probe*	Cy5-ATCGTTTACCGACTTTAG- BHQ2		[37]
***Necator americanus***	Fwd	CTGTTTGTCGAACGGTACTTGC	ITS-2	[37]
	Rev	ATAACAGCGTGCACATGTTGC		[37]
	Probe*	FAM-CTGTACTACGCATTGTATAC-BHQ1		[37]

*Minor groove binding probes; Fwd: forward primer; Rev: reverse primer; BHQ1: black hole quencher 1; BHQ2: black hole quencher 2; ITS-1: internal transcribed spacer 1; 18S: 18S ribosomal RNA gene; ITS-2: internal transcribed spacer 2

### Stool preservation experiment

#### Selection of samples

In April 2016, a total of 140 stool samples were collected from children of two primary schools in Jimma Town, with the ultimate aim to have 20 stool samples with at least 150 EPG for two STH species. Stool samples were microscopically screened applying a single Kato-Katz thick smear as previously described (WHO, 1992). To avoid clearance of hookworm eggs, all smears were examined within 30–60 min for the presence of STH eggs. The number of STH eggs was counted as per STH species basis and multiplied by 24 to obtain the FECs in EPG.

#### Stool preservation

After homogenization, three aliquots of 0.5 gram from each of the selected samples were preserved in 1 ml of 96% ethanol, three aliquots in 1 ml of 5% potassium dichromate and three aliquots in 1 ml of RNA*later* (Invitrogen). All samples were preserved within 6 hours after the stool collection. Except during the shipment of the samples, all aliquots were stored at 4°C until DNA extraction. To avoid any systematic error (e.g., the first aliquot being assigned to the same preservative and duration of preservation), we randomized the aliquots to one of the three preservatives and time points of DNA extraction. At 65, 245 and 425 days of preservation, one of the three aliquots was subjected to DNA extraction.

#### DNA extraction and qPCR

DNA of preserved stool samples were extracted using the in-house protocol of the Laboratory for Medical Microbiology and Immunology, Elisabeth-TweeSteden Hospital, Tilburg (The Netherlands). Briefly, 250 μl of stool suspension was placed in an Eppendorf tube and washed by centrifugation at 8,500 g for 1 min. Subsequently, the supernatant was aspirated and discarded. A total volume of 1 ml PBS was added to the pellet, which was again vortexed and centrifuged at 8,500 g for 1 min. Then the supernatant was once more discarded. Subsequently, 500 μl of 2% polyvinylpyrrolidone (PVPP, Sigma) was added to prevent inhibition in downstream qPCR steps, and the suspension was transferred to Green Bead tubes (Roche). A freeze-thaw cycle was performed by placing the tubes at -80°C for 30 min. After thawing, mechanical disruption of the sample was performed by placing the tubes in the MagNA Lyser (Roche) for 1 min at 3,000 rpm. After a short spin, 500 μl of ATL buffer (Qiagen) containing 50 μl of Proteinase K (Qiagen) was added and placed at 55°C for 2 h. Subsequently, samples were placed in the automated QiaSymphony platform for purification. Finally, extracted DNA from each preservative and each time point was used as a template in qPCR assays for the quantification of DNA of *A*. *lumbricoides*, *T*. *trichiura* and hookworms (*N*. *americanus* and *A*. *duodenale*) using the primers described in **Table 1**.

### Data analysis

#### DNA extraction protocols

The performance of the different extraction protocols was assessed by comparing the sensitivity and the DNA concentration of the different STHs expressed as GE/ml. The sensitivity was determined using the principle of the composite reference standard method [38] as the gold standard, which in our case classifies a stool sample as positive for a STH species if at least one egg was detected applying microscopy or one of the four extraction protocols has a downstream positive qPCR for that particular STH species. For the DNA concentration, the geometric mean was applied as a summary statistic. We explored the sensitivity and DNA concentration for each of the four DNA extraction protocols separately. We determined the sensitivity across the different levels of infection intensity and the Pearson’s coefficient between the log transformed DNA concentration and the log transformed FECs.

Significant differences in sensitivity and DNA concentration across DNA extraction protocols were assessed applying generalized linear mixed effect models. For the sensitivity, the ‘glmer’ function in R was used, incorporating the binary qPCR test (positive or negative for that particular target) as dependent variable and STH species (2 levels: *T*. *trichiura* and *N*. *americanus*), the DNA extraction kit (2 levels: QIAamp DNA Stool Mini kit and DNeasy Blood & Tissue kit), the inclusion of a bead beating step (2 levels: yes and no) and log transformed FECs as potential predicting variables. For the DNA concentration, the ‘lmer’ function in R was used, incorporating the log transformed DNA concentration of that particular target as dependent variable and the aforementioned potential predicting variables. In both analyses, only those samples that were found positive based on the composite reference standard method were incorporated in the analysis. In addition, samples containing DNA of multiple STHs were considered as separate samples, one for each STH species. The level of significance was set at *p* <0.05.

#### Stool preservation

We assessed the impact of different preservatives, duration of preservation and FECs by comparing the sensitivity and the DNA concentration of the different STHs expressed as GE/ml applying the aforementioned methodologies. For the sensitivity, a stool sample was classified as positive for a STH species if at least one egg was detected applying microscopy or one of the preservatives had a downstream positive qPCR at least one time point of DNA extraction for that particular STH species. For the generalized linear mixed effect models, the STH species (3 levels: *A*. *lumbricoides*, *T*. *trichiura* and *N*. *americanus*), the preservative (3 levels: 96% ethanol, RNA*later* and 5% potassium dichromate), duration of preservation expressed in days (continuous variable) and the log transformed FECs were evaluated as potential predicting variables. The level of significance was set at *p* <0.05. The raw data of the two different experiments is made available in **S2 File**.

## Results

### Comparison of the four DNA extraction protocols

**Fig 1:**
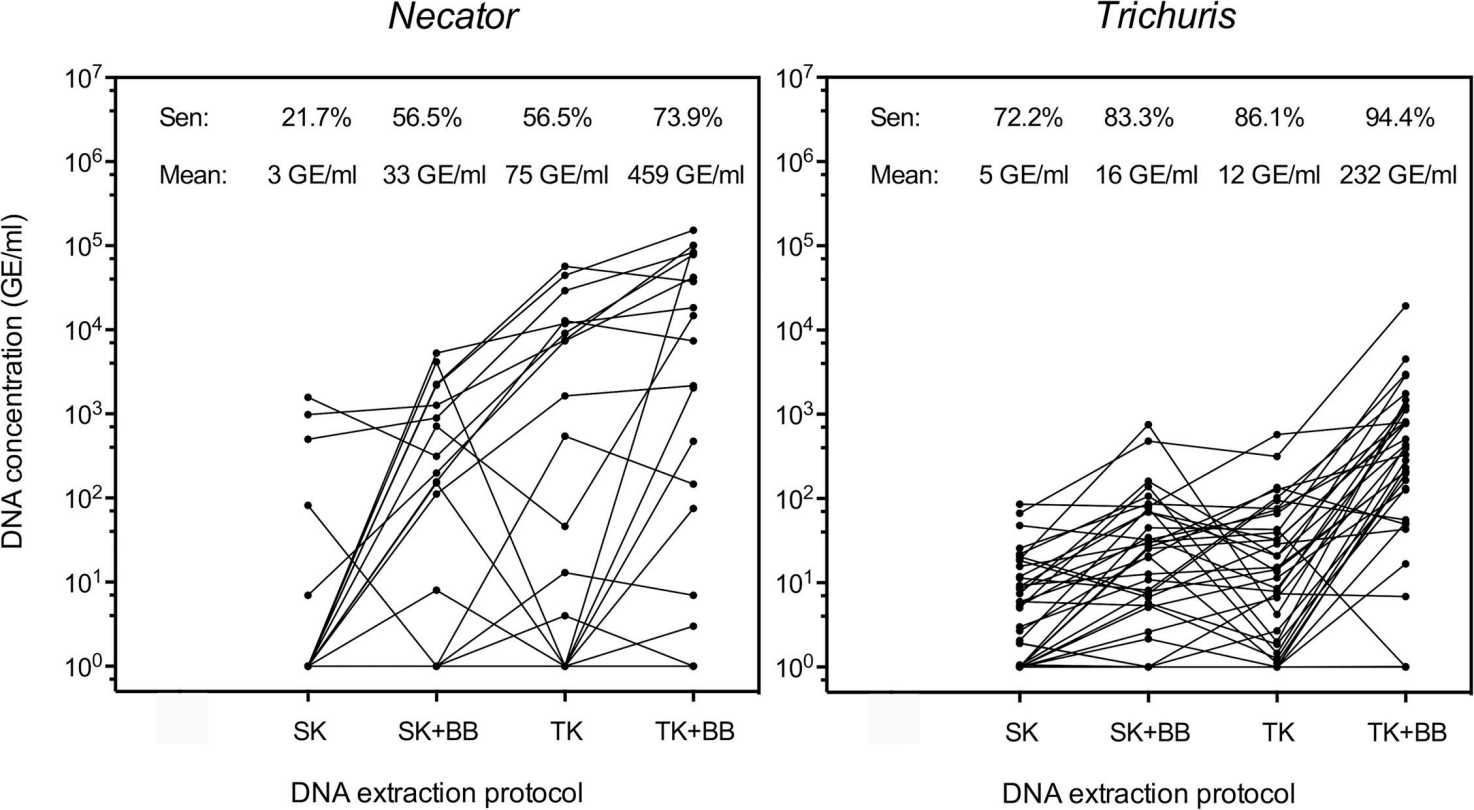
The sensitivity and DNA concentration across four DNA extraction protocols for *Trichuris* and *Necator*. The left panel represents the sensitivity (sen) and geometric mean of DNA concentration expressed as genome equivalents per ml of DNA extract (mean; GE/ml) for 20 *Necator americanus* samples preserved in 96% ethanol and extracted by four DNA extraction protocols. The DNA extraction protocols include the QIAamp DNA Stool Mini kit without (SK) and with bead beating (SK + BB), DNeasy Blood & Tissue kit without (TK) and without bead beating (TK + BB). The right panel represents the same parameters across 36 *Trichuris trichiura* samples preserved in 96% ethanol. Each line represents a sample.

Eggs and/or DNA of *T*. *trichiura* and *N*. *americanus* were detected in 36 and 23 samples, respectively. **Fig 1** illustrates the sensitivity and DNA concentration for each of the four DNA extraction protocols for both *T*. *trichiura* and *N*. *americanus*. The sensitivity when applying the DNeasy Blood & Tissue kit was higher compared to the QIAamp Stool Mini kit for both STH species, and this independent of the inclusion of bead beating. Similar trends were observed for DNA concentration. The measured DNA concentrations were higher when the DNeasy Blood & Tissue kit was used and when a bead beating step was applied. For both STHs, we also explored the variation in sensitivity across the FECs (See **Table 2**). In general, the sensitivity increased when the FECs increased and this increase in sensitivity was proportionally more pronounced when QIAamp Stool Mini kit or no bead beating was applied.

**Table 2:** The sensitivity across four DNA extraction protocols for different levels of *Trichuris* and *Necator* infections. The intensity of infection was classified as low (*T*. *trichiura*: fecal egg count (FEC) <1,000 eggs per gram of stool (EPG); *N*. *americanus*: FEC <2,000 EPG) and as moderate-to-heavy (*T*. *trichiura*: FEC ≥1,000 EPG; *N*. *americanus*: FEC ≥2,000 EPG). The zero FECs, represent subjects for which no eggs were found applying McMaster.

		N	QIAamp DNA Stool Mini kit		DNeasy Blood & Tissue kit	
			Without bead beating (%)	With bead beating (%)	Without bead beating (%)	With bead beating (%)
***Trichuris trichiura***						
	Zero FECs	15	66.7	73.3	73.3	86.7
	Low	11	54.5	81.8	90.9	100
	Moderate-to-heavy	10	100	100	100	100
***Necator americanus***						
	Zero FECs	9	22.2	44.4	44.4	66.7
	Low	14	21.4	64.3	64.3	78.6

For *T*. *trichiura*, there was a positive correlation between the FECs and the DNA concentration for each of the four DNA extraction protocols (See **S3 File**), the correlation being higher when a bead beating step was applied (QIAamp DNA Stool Mini kit: 0.39 (*p* = 0.03) *vs*. 0.47 (*p* = 0.004); DNeasy Blood & Tissue kit: 0.32 (*p* = 0.07) *vs*. 0.64 (*p* <0.001)). Similar patterns were observed for *N*. *americanus* (QIAamp DNA Stool Mini kit: 0.01 (*p* = 0.96) *vs*. 0.35 (*p* = 0.10); DNeasy Blood & Tissue kit: 0.23 (*p* = 0.28) *vs*. 0.31 (*p* = 0.14); **S4 File**), but the correlation coefficients were not significant for all DNA extraction protocols.

The outcome of the general linear mixed effect regression models confirmed significant differences in sensitivity and DNA recovery across both STHs, DNA extraction protocols and FECs. The odds of detecting a case of *N*. *americanus* was approximately 18.1 (95% confidence intervals (95%CI): 3.9–84.6, *p* <0.001) times lower than the odds for *T*. *trichiura*. Applying the DNeasy Blood & Tissue kit increased the odds with a factor 5.6 (95%CI: 2.2–14.1, *p* <0.001) compared to the QIAamp DNA Stool Mini kit. Inclusion of a bead beating step further improved the detection of cases, the odds being 4.8 times higher (95%CI: 1.9–11.8, *p* <0.001) than when bead beating was not included. An increase in FEC of 1 EPG increased the odds ratios 1.3 times (95%CI: 1.0–1.6, *p* = 0.02). The two-way interactions between STH, DNA extraction kit, bead beating and FECs were not significant.

The DNA concentration increased approximately 6.3 (95%CI: 3.5–11.5) times when the DNeasy Blood & Tissue kit was used instead of the QIAamp DNA Stool Mini kit. A bead beating step increased the DNA concentration 3.6 (96%CI: 1.8–7.4) times. There was no significant difference in DNA recovery across *N*. *americanus* and *T*. *trichiura*, but the impact of the DNA extraction kit on DNA recovery was different for these STHs. Compared to *T*. *trichiura*, the DNA recovery gain when applying the DNeasy Blood & Tissue kit instead of QIAamp DNA Stool Mini kit was 2.8 (96%CI: 1.1–7.4) times higher for *N*. *americanus*. An interaction was also observed between FECs and the bead beating. When the FEC increases with 1 EPG, bead beating resulted in the DNA concentration that was 1.2 (95%CI: 1.1–1.4) times higher compared to when no bead beating was done.

### Comparison of the different stool preservatives and period of preservation

**Fig 2:**
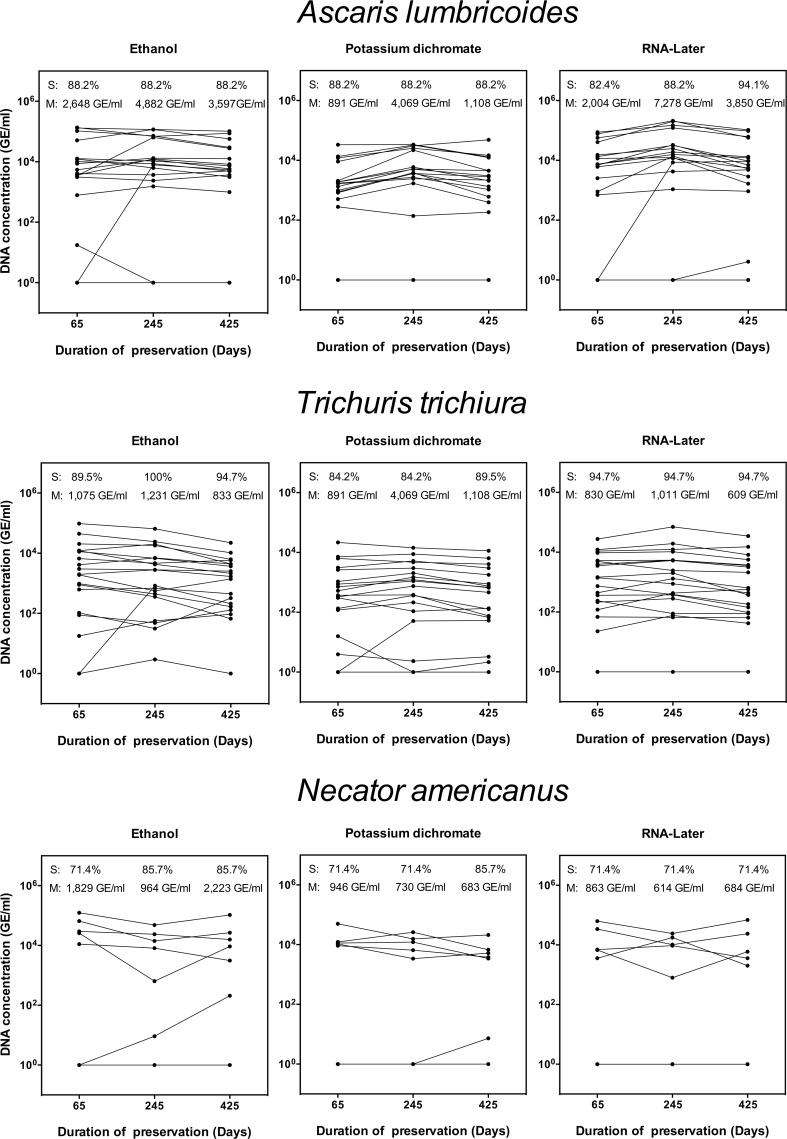
The sensitivity and DNA concentration across three preservatives and three preservation times. The line graphs represent the sequential differences in sensitivity (Sen) and geometric mean (mean) of DNA concentration expressed genome equivalents/ml (GE/ml) of DNA over time (65, 125 and 425 days) for 17 *Ascaris lumbricoides*, 19 *Trichuris trichiura* and 7 *Necator americanus* samples stored in three preservatives. The preservatives include 96% ethanol (left plot), 5% potassium dichromate (middle graph) and RNA*later* (right graph). Each line represents a sample.

Eggs and/or DNA of *A*. *lumbricoides*, *T*. *trichiura* and *N*. *americanus* were detected in 17, 19 and 7 samples, respectively. **Fig 2** summarizes the sensitivity and DNA concentration for each of the preservatives for *A*. *lumbricoides*, *T*. *trichiura* and *N*. *americanus* over time. In general, there were only minor differences in sensitivity and DNA concentration over time and DNA concentration in samples preserved in potassium dichromate where lower compared to that found in sample preserved in ethanol and RNA*later*. As illustrated in **S5 File**, the sensitivity generally increased with increasing FECs for the three STH species. The gain in detecting cases was more pronounced for *T*. *trichiura* and potassium dichromate. For all three STH species, there was a significant correlation between the FECs and the DNA concentration for each of the three preservatives (See **S6 File**), the correlation being high (Pearson’s correlation coefficient (R) >0.79) for each of the preservatives and STH species.

The outcome of the regression models indicated a significant difference in sensitivity between STHs and FECs, but did not reveal any significant difference between preservatives and duration of preservation. Compared to *A*. *lumbricoides* (reference), the odds of correctly classifying a case were 2.1 times (95%CI: 1.0; 4.5, *p* = 0.06) lower for *N*. *americanus*, though only marginally significant. For *T*. *trichiura*, there was no significant difference in odds ratio. A significant difference in DNA concentration was observed between STHs, preservatives and FECs, but not for duration of preservation. Compared to *A*. *lumbricoides* (reference), the DNA concentration for *N*. *americanus* was 8.8 times (95%CI: 1.5–52.7) lower. The DNA concentration increased with a factor 1.5 (95%CI: 1.2–1.8) per increase of 1 EPG. In addition, there was a significant interaction between FECs, and both STHs and preservatives, indicating that the gain in DNA concentration for an equal increase FEC is different across STHs and preservatives. When compared to *A*. *lumbricoides* (reference), an increase in FEC with 1 EPG (reference) resulted in 1.4 (95%CI: 1.0–1.9) higher DNA concentration for *N*. *americanus* and 1.4 (95%CI: 1.1–1.7) times lower DNA concentration for *T*. *trichiura*. When compared to the DNA concentration of samples preserved ethanol (reference), an increase in FEC with 1 EPG resulted in 1.3 less DNA when preserved in potassium dichromate (95%CI: 1.0–1.7) and RNA-later (95%CI: 1.1–1.6).

## Discussion

Research and clinical laboratories are increasingly using NAATs for both detection and quantification of STHs in stool. However, any DNA based examination requires DNA extraction method that yields sufficient and qualitative DNA. In the current study, we assessed the performance of the different extraction and preservation protocols by comparing the sensitivity and the DNA concentration (expressed as GE/ml) of the different STHs. To our knowledge, we are the first to compare the preservation of stool samples over a period of 14 months in three widely used preservatives for STHs, i.e. ethanol, potassium dichromate and RNA*later*.

### DNeasy Blood & Tissue kit outcompetes QIAamp DNA Stool Mini

The DNA extraction experiment indicated that extracting DNA applying the DNeasy Blood & Tissue kit improved the sensitivity and DNA concentration for both *T*. *trichiura* and *N*. *americanus*. Moreover, the gain in DNA concentration significantly varied across kits, with the DNA recovery when using the DNeasy Blood & Tissue kit being higher for *N*. *americanus* than for *T*. *trichiura*. The relatively poor performance of the QIAamp DNA Stool Mini kit is rather unexpected, particularly when this kit is designed for extracting DNA from stool, for example by inclusion of a inhibitor removal step. Although it remains difficult to identify any other differences in both protocols (e.g. recipe of different buffers is not known; ASL in stool kit *vs*. ATL buffer in tissue kit), there is a clear difference in both time and temperature at which the samples are lysed (stool kit: 10 min at 70°C *vs*. tissue kit: 2 hours at 55°C). In addition to a lesser performance, the QIAamp DNA Stool Mini kit is also more expensive than the DNeasy Blood & Tissue kit (€322 *vs*. €201 for 50 samples, prices obtained from the company website) [39,40] and requires more operational steps (e.g. adding and removal of the inhibitEX tablet). In the present study we only tested two kits from the same company, but there is a plethora of commericial DNA extractions kits (e.g. DNease PowerSoil kit (fomer PowerSoil kit), FastDNA SPIN Kit and MagnaPure Roche kit; (Cools et al., under review), and hence it remains unclear whether these kits outcompete the DNeasy Blood & Tissue kit.

### Bead beating is a crucial step in any DNA extraction protocol

The DNA extraction experiment clearly showed that a bead beating step too has an important impact on the sensitivity and DNA recovery. This is in line with previous studies that compared DNA extraction protocols with and without bead beating for the detection of parasites, including but not limited to human STHs (*A*. *lumbricoides* [33], *A*. *suum* [41], *A*. *duodenale* [33], *Necator americanus* [33], *T*. *trichiura* [33,41], *Ostertagia ostertagi* [42], *Echinococcus multilocularis* [43,44], *Toxocara canis*, *Toxocara catis* and *Toxoascaris leonina* [45]). In addition, the benefit of bead beating was more pronounced when FECs were higher, which might be explained by the increased likelihood of contact of beads with eggs (being higher when more eggs are present for same number of beads), and suggests that a higher concentration of beads might be recommended when FECs are low. The current and previous studies have used different types of beads such as glass [46], garnet [33], zirconium [41], ceramic [42] and steel [45], but the impact of these on the sensitivity of and DNA concentration too remains to be elucidated.

### Ethanol is a cheap and reliable preservative for long-term storage for STHs

We found that ethanol and RNA*later* preserved stool samples yielded higher DNA concentrations of *A*. *lumbricoides*, *T*. *trichiura* and *N*. *americanus* compared to potassium dichromate although there were no significant differences in sensitivity across preservatives. Furthermore, for each of the preservatives we demonstrated limited variation in DNA concentration over the evaluated time period. This justifies the use of both ethanol, potassium dichromate and *RNA*later for long time storage. These findings are in line with previous studies comparing preservation of *N*. *americanus* (ethanol, potassium dichromate and *RNA*later over 60 days) [28] and *Giardia duodenalis* cysts (ethanol and potassium dichromate over three months) [47]. On the contrary, Kuk and Cetinkaya (2012) suggested that potassium dichromate was a better preservative for *G*. *duodenalis* trophozoites compared to 75% ethanol (over four weeks), but conclusions were based on a small sample size (n = 5) [48]. Ethanol has some important advantages over the other two preservatives. First, the results highlight that the net gain in DNA concentration compared to other preservatives increased when FEC are higher, suggesting that it is better preservative. Second, ethanol is easily accessible in resource-limited settings, where e.g. the commercial *RNA*later and potassium dichromate can be difficult to obtain. Moreover, ethanol is approximately two times and over 100 times cheaper compared to potassium dichromate (5%) and *RNA*later, respectively. The cost of 100 ml ethanol, 100 ml potassium dichromate (5%) and 100 ml *RNA*later were estimated to be €1, €1.8 and €150, respectively.

### Limitations of the study

This study has six important limitations, which needs to be considered when both interpreting extrapolating the results. First, we only evaluated a restricted number of DNA extraction (DNA extraction kit and beads) and preservative protocols, focusing on DNA extraction protocols previously used by our laboratory and widely used preservatives. Consequentially, extrapolation to any other combination of DNA and preservation protocol should be done with care. Second, the two experiments were conducted on two different sets of stool samples at different time points applying a different microscopic method for screening cases (DNA extraction experiment: McMaster and preservation experiment: Kato-Katz thick smear) and a slightly adapted DNA extraction protocol on the selected samples (e.g. manual vortexing *vs*. automated bead beating). Although this study is indeed not standardized across both experiments, this difference in methodology across experiments has no impact on the conclusions drawn from the separate experiments. Third, we did not include a non-preservation control (extraction of fresh samples at time of collection) or a standardized reference (e.g. freezing of samples) in our experiments. Hence, we cannot exclude any DNA degradation with absolute certainty. Inclusion of a non-preservative control or a standard reference was logistically difficult. All samples needed to be shipped to The Netherlands for further molecular analysis, which impeded extraction of all samples at collection (fresh samples would have been extracted with a slightly different DNA extraction protocol), required strict measurements to adhere to safety regulations and to ensure cold chain throughout the transfer of material. Moreover, it is important to highlight that previous studies indicated that analysis of samples preserved in ethanol provide a level of performance that was at least equal to that of samples immediately frozen after collection [33]. This underscores that ethanol-preserved samples too could serve as valid standard reference, which on top is more feasible under field conditions. Fourth, we stored the preserved samples at 4°C under controlled conditions (except during shipment of the samples). Although there is an increased storage capacity in STH endemic countries, these conditions and thus the results might not be representative for all laboratory settings. Fifth, due to the low endemicity we were not able to include sufficient cases of *N*. *americanus*, and hence more research is required to confirm our findings. In addition to this, we are not able to draw any conclusions on the other hookworm species (*A*. *duodenale* and *A*. *ceylanicum*).

In conclusion, DNeasy Blood & Tissue kit DNA extraction protocol with preceded bead beating maximized the extraction of STH DNA in stool in our study. Ethanol was found to be the most cost-effective preservative. Given the clear benefit of bead beating and our validation of ethanol for (long-term) preservation, we recommend that these aspects of the protocol should be adopted by any stool sampling and DNA extraction protocol for downstream NAAT-based detection and quantification of STHs.

## Supporting information

S1 File A Detailed Standard Operating Procedure on How to Prepare Standard Dilution Series of Genomic DNA of *Ascaris lumbricoides*, *Trichuris trichiura* and *Necator americanus*. (PDF)

S2 File The Data of Both the DNA Extraction and Preservation Experiment. The raw data of the DNA and preservation experiment can be found in the worksheets ‘DNA extraction’ and ‘Preservation’, respectively. The worksheet ‘Legend’ explains the headers (variables) in across both data sets.
(XLSX)

S3 File Agreement in Fecal Egg Counts and DNA Concentration Across Four DNA Extraction Protocols for *Trichuris*. The fecal egg counts are expressed as eggs per gram of stool (EPG), whereas the DNA concentration is expressed as genome equivalents per ml (GE/ml). **‘**R’ represents the Pearson’s correlation coefficient.
(PDF)

S4 File Agreement in Fecal Egg Counts and DNA Concentration across Four DNA Extraction Protocols for *Necator*. The fecal egg counts are expressed as eggs per gram of stool (EPG), whereas the DNA concentration is expressed as genome equivalents per ml (GE/ml). **‘**R’ represents the Pearson’s correlation coefficient.
(PDF)

S5 File The Sensitivity for Three Preservatives Protocols for Different Levels of Soil-Transmitted Helminth Infections. The intensity of infection was classified as low (*Ascaris lumbricoides*: fecal egg count (FEC) <5,000 eggs per gram of stool (EPG)*; Trichuris trichiura*: FEC <1,000 EPG; *Necator americanus*: FEC <2,000 EPG) and as moderate-to-heavy (*Ascaris lumbricoides*: FEC ≥5,000 EPG; *Trichuris trichiura*: FEC ≥1,000 EPG; *Necator americanus*: FEC ≥2,000 EPG). The zero FECs, represent subjects for which no eggs were found applying Kato-Katz thick smear, but for which at least on preservation protocol resulted in a positive qPCR. The sample size equals to the number (N) of subjects multiplied by the number of time points at which DNA is extracted (3 time points).
(DOCX)

S6 File Agreement in Fecal Egg Counts and DNA Concentration across Three Preservation Protocols for Soil-Transmitted Helminths. The scatterplot illustrate the agreement in fecal egg counts (FECs; expressed as eggs per gram of stool (EPG) and the DNA concentration (expressed as genome equivalents per ml (GE/ml)) across ethanol, potassium dichromate and RNA*later* for *Ascaris lumbricoides* (top graphs), *Trichuris trichiura* (middle graphs) and *Necator americanus* (bottom graphs).’R’ represents the Pearson’s correlation coefficient.
(PDF)
