# Supplementary material for: Comparison of four DNA extraction and three preservation protocols for the molecular detection and quantification of soil-transmitted helminths in stool
Source: PLoS Negl Trop Dis. 2019 Oct 28;13(10):e0007778. doi: 10.1371/journal.pntd.0007778 (PMC6837582; doi:10.1371/journal.pntd.0007778)
Supplement: S1 File — (PDF) [file pntd.0007778.s001.pdf]

Authors:
Piet Cools & Kenneth Hofkens

Approved by:
Jaco Verweij & Bruno Levecke

SOP 20: Preparation of standard dilution series of genomic DNA of *Ascaris lumbricoides*, *Trichuris trichiura* and *Necator americanus*

1. Purpose

This SOP describes the procedures to generate standard dilution series of genomic DNA (gDNA) of *Ascaris lumbricoides*, *Trichuris trichiura* and *Necator americanus*. These standard dilution series are needed **(i)** for the generation of a standard curve that is needed for the absolute quantification of *A. lumbricoides*, *T. trichiura* and *N. americanus* by means of qPCR (see SOP 21) and **(ii)** to determine the limit of detection and the limit of quantification of these assays (see SOP 22).

2. Biological material, equipment and reagents

- Worm/larval material (See Table 1).
- Equipment & reagents needed for DNA extraction of worm/larval material (See SOP 19).
- Qubit 2.0 fluorometer (ThermoFisher Scientific)
- Eppendorf tubes
- Molecular water
- Filter pipette tips and pipettes

Table 1. Origin and the nature of worm material.

**Species**	**Nature**	**Origin**
Ascaris lumbricoides	Heads of 2 adult worms	Expulsion study in Jimma Town, Ethiopia
Trichuris trichiura	2 complete adult worms	Expulsion study in Copenhagen, Denmark **(Ref. 1)**
Necator americanus	Larvae (pellet of ±2 mm diameter)	Laboratory strain at Swiss Tropical & Public Health Institute

3. Procedures

3.1. Preparation of the standard dilution series

1. Extract the DNA from the biological material listed in Table 1 according to Starworms SOP 19 ‘DNA extraction and purification of soil-transmitted helminth worms and larvae’. Label the tube as ‘0’ (referring to dilution 10^0, which is the undiluted DNA extract) followed by the species name (**AL**, *Ascaris lumbricoides*; **TT**, *Trichuris trichiura*; **NA**, *Necator americanus*).

2. Measure the concentration of each DNA extract **three times** using the Qubit 2.0 fluorometer and calculate the average DNA concentration.

3. For each DNA extract, make a tenfold dilution series of eight dilutions of 100 µl as follows:

   a. Label the seven tubes as ‘-1’, ‘-2’, ‘-3’, etc. (referring to dilutions 10^{-1}, 10^{-2}, 10^{-3}, etc.), followed by the species (**AL**, *Ascaris lumbricoides*; **TT**, *Trichuris trichiura*; **NA**, *Necator americanus*).

   b. Pipet 90 µl of molecular water in each of these seven Eppendorf tubes using filter tips.

   c. Pipet 10 µl of the original extract (‘dilution 0’) in the dilution ‘-1’ using filter tips. Gently resuspend by pipetting up and down a few times.

   d. Pipet 10 µl of this dilution in the next dilution ‘-2’ and gently resuspend by pipetting up and down a few times. Continue making the rest of the dilution series in a similar way.

4. Store the standard dilution series at – 20 °C.

3.2. Calculation of the DNA concentrations of the standard dilution series

1. The DNA concentration of each DNA extract (expressed in genome equivalents/ml (GE/ml)) is calculated using the following equation:

Genomic DNA concentration (GE/ml) = DNA concentration extract (ng/ml) / mass genome (ng)

The mass of the different STH genomes is listed in Table 2.

2. The genomic concentration of the different dilutions of each series is obtained by dividing the dilution factor of the different dilutions.

Table 2. Genome characteristics of the STH.

Species	Genome size (Mb)	GC% content genome	Mass genome (ng)*	Reference
Ascaris lumbricoides	316.98	37.5	$3{,}25\ 10^{-4}$	2
Trichuris trichiura	75.18	42.2	$7{,}71\ 10^{-5}$	3
Necator americanus	244.01	40.2	$2{,}50\ 10^{-4}$	4

Legend: Mb, mega base pairs (1 Mb equals 10^6 base pairs). *Calculated from the published genome size and GC% content as described in Addendum.

4. Addendum

The mass of the genome of the different species was calculated from the published genomes of the different species (Table 1) using the equation below:

$$\left(\frac{(\text{n° GC bp} \times 618.4\ \text{g/mol})}{N_A} + \frac{(\text{n° AT bp} \times 617.4\ \text{g/mol})}{N_A} \right) \times 10^9$$

In this equation,

- n° GC bp = the number of CG base pairs, calculated from the published genome size and the published GC% (Table 1);
- n° AT bp = the number of AT base pairs, calculated from the published genome size and the published GC% (Table 1);
- 618.4 g/mol = the molecular mass of 1 GC base pair, expressed in grams per mol (Ref. 4);
- 617.4 g/mol = the molecular mass of 1 AT base pair, expressed in grams per mol (Ref. 4);
- N_A = the constant of Avogadro, which equals $6.022 \times 10^{23}\ \text{mol}^{-1}$

5. References

1. Hansen EP, et al. Faecal egg counts and expulsion dynamics of the whipworm, Trichuris trichiura following self-infection. J Helminthol. 2016 May;90(3):298-302.
2. https://parasite.wormbase.org/species.html
3. Foth et al. Whipworm genome and dual-species transcriptome analyses provide molecular insights into an intimate host-parasite interaction. Nature Genetics. 2014 Jul;46(7):693-700.
4. Tang et al. Genome of the human hookworm *Necator americanus*. Nature Genetics. 2014 Mar;46(3):261-269.
5. Liquid Chromatography-Mass Spectrometry of Nucleic Acids. Herbert Oberacher and Walther Parson. In: *Medical BioMethods Handbook. 2005.* Edited by John M. Walker and Ralph Rapley. Humana Press, Totowa, New Jersey.
