# Supplementary material for: Comparison of four DNA extraction and three preservation protocols for the molecular detection and quantification of soil-transmitted helminths in stool
Source: PLoS Negl Trop Dis. 2019 Oct 28;13(10):e0007778. doi: 10.1371/journal.pntd.0007778 (PMC6837582; doi:10.1371/journal.pntd.0007778)
Supplement: S4 File — The fecal egg counts are expressed as eggs per gram of stool (EPG), whereas the DNA concentration is expressed as genome equivalents per ml (GE/ml). ‘R’ represents the Pearson’s correlation coefficient. (PDF) [file pntd.0007778.s004.pdf]

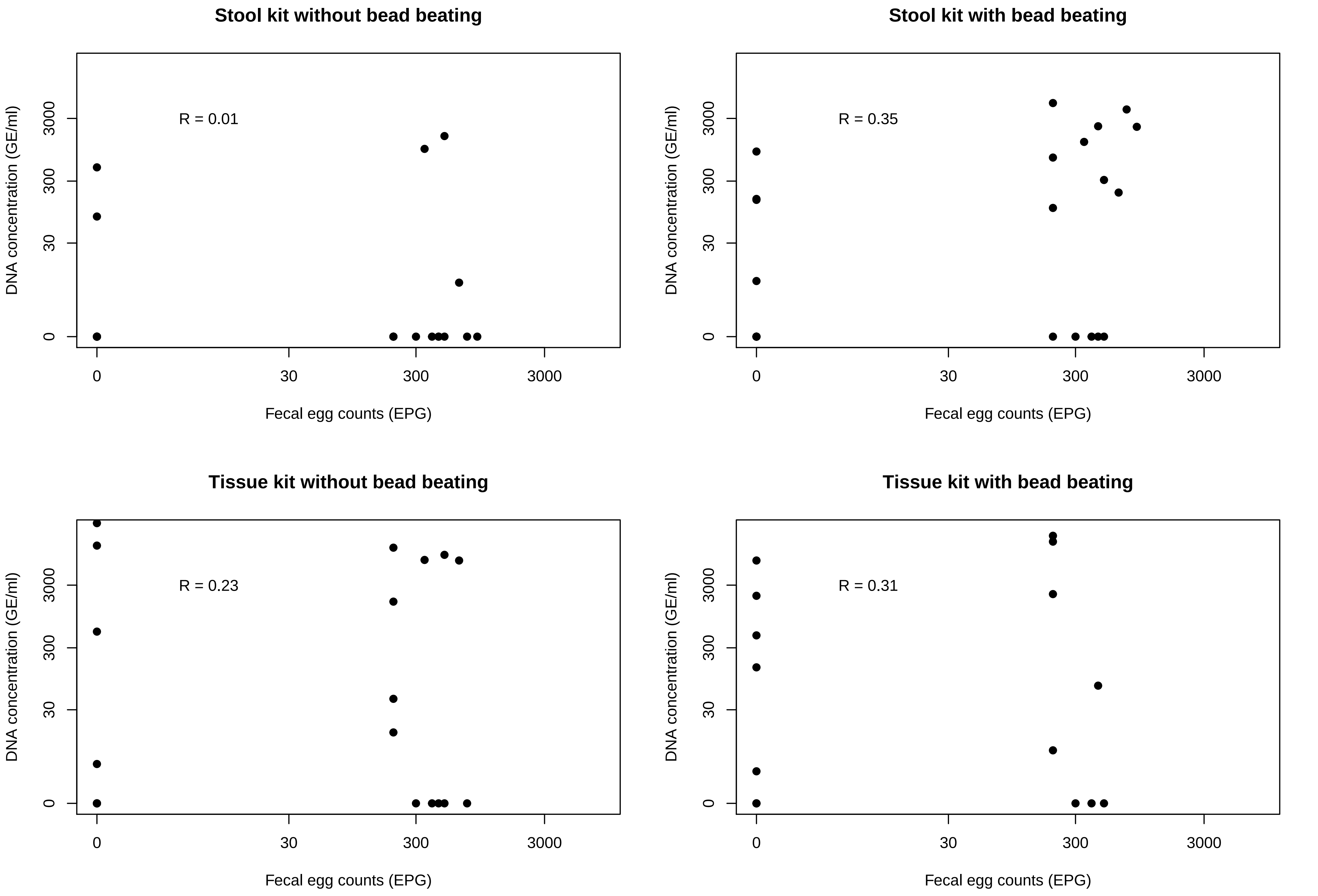
Stool kit without bead beating
R = 0.01
Stool kit with bead beating
R = 0.35
Tissue kit without bead beating
R = 0.23
Tissue kit with bead beating
R = 0.31
DNA concentration (GE/ml)
0
30
300
3000
Fecal egg counts (EPG)
