# Supplementary material for: Comparison of four DNA extraction and three preservation protocols for the molecular detection and quantification of soil-transmitted helminths in stool
Source: PLoS Negl Trop Dis. 2019 Oct 28;13(10):e0007778. doi: 10.1371/journal.pntd.0007778 (PMC6837582; doi:10.1371/journal.pntd.0007778)
Supplement: S5 File — The intensity of infection was classified as low (Ascaris lumbricoides: fecal egg count (FEC) <5,000 eggs per gram of stool (EPG); Trichuris trichiura: FEC <1,000 EPG; Necator americanus: FEC <2,000 EPG) and as moderate-to-heavy (Ascaris lumbricoides: FEC ≥5,000 EPG; Trichuris trichiura: FEC ≥1,000 EPG; Necator americanus: FEC ≥2,000 EPG). The zero FECs, represent subjects for which no eggs were found applying Kato-Katz thick smear, but for which at least on preservation protocol resulted in a positive qPCR. The sample size equals to the number (N) of subjects multiplied by the number of time points at which DNA is extracted (3 time points). (DOCX) [file pntd.0007778.s005.docx]

**Supplementary Info S4. The sensitivity for three preservatives protocols for different levels of soil-transmitted helminth infections.** The intensity of infection was classified as low (*Ascaris*: fecal egg count (FEC) <5,000 eggs per gram of stool (EPG)*; Trichuris*: FEC <1,000 EPG; *Necator*: FEC <2,000 EPG) and as moderate-to-heavy (*Ascaris*: FEC ≥5,000 EPG; *Trichuris*: FEC ≥1,000 EPG; *Necator*: FEC ≥2,000 EPG). The zero FECs, represent subjects for which no eggs were found applying Kato-Katz thick smear, but for which at least on preservation protocol resulted in a positive qPCR. The sample size equals to the number (N) of subjects multiplied by the number of time points at which DNA is extracted (3 time points).

|  |  | N subjects x N time points | Ethanol (%) | Potassium dichromate (%) | RNA*later®* (%) |
| --- | --- | --- | --- | --- | --- |
| *Ascaris* |  |  |  |  |  |
|  | Zero FECs | 1 x 3 | 100 | 100 | 100 |
|  | Low | 8 x 3 | 100 | 100 | 95.8 |
|  | Moderate-to-heavy | 6 x 3 | 94.4 | 100 | 100 |
| *Trichuris* |  |  |  |  |  |
|  | Zero FECs | 4 x 3 | 83.3 | 41.7 | 75.0 |
|  | Low | 10 x 3 | 96.7 | 96.7 | 100 |
|  | Moderate-to-heavy | 5 x 3 | 100 | 100 | 100 |
| *Necator* |  |  |  |  |  |
|  | Zero FECs | 2 x 3 | 33.3 | 16.7 | 0 |
|  | Low | 5 x 3 | 100 | 100 | 100 |
