# Supplementary material for: Comparison of four DNA extraction and three preservation protocols for the molecular detection and quantification of soil-transmitted helminths in stool
Source: PLoS Negl Trop Dis. 2019 Oct 28;13(10):e0007778. doi: 10.1371/journal.pntd.0007778 (PMC6837582; doi:10.1371/journal.pntd.0007778)
Supplement: S6 File — The scatterplot illustrate the agreement in fecal egg counts (FECs; expressed as eggs per gram of stool (EPG) and the DNA concentration (expressed as genome equivalents per ml (GE/ml)) across ethanol, potassium dichromate and RNAlater for Ascaris lumbricoides (top graphs), Trichuris trichiura (middle graphs) and Necator americanus (bottom graphs).’R’ represents the Pearson’s correlation coefficient. (PDF) [file pntd.0007778.s006.pdf]

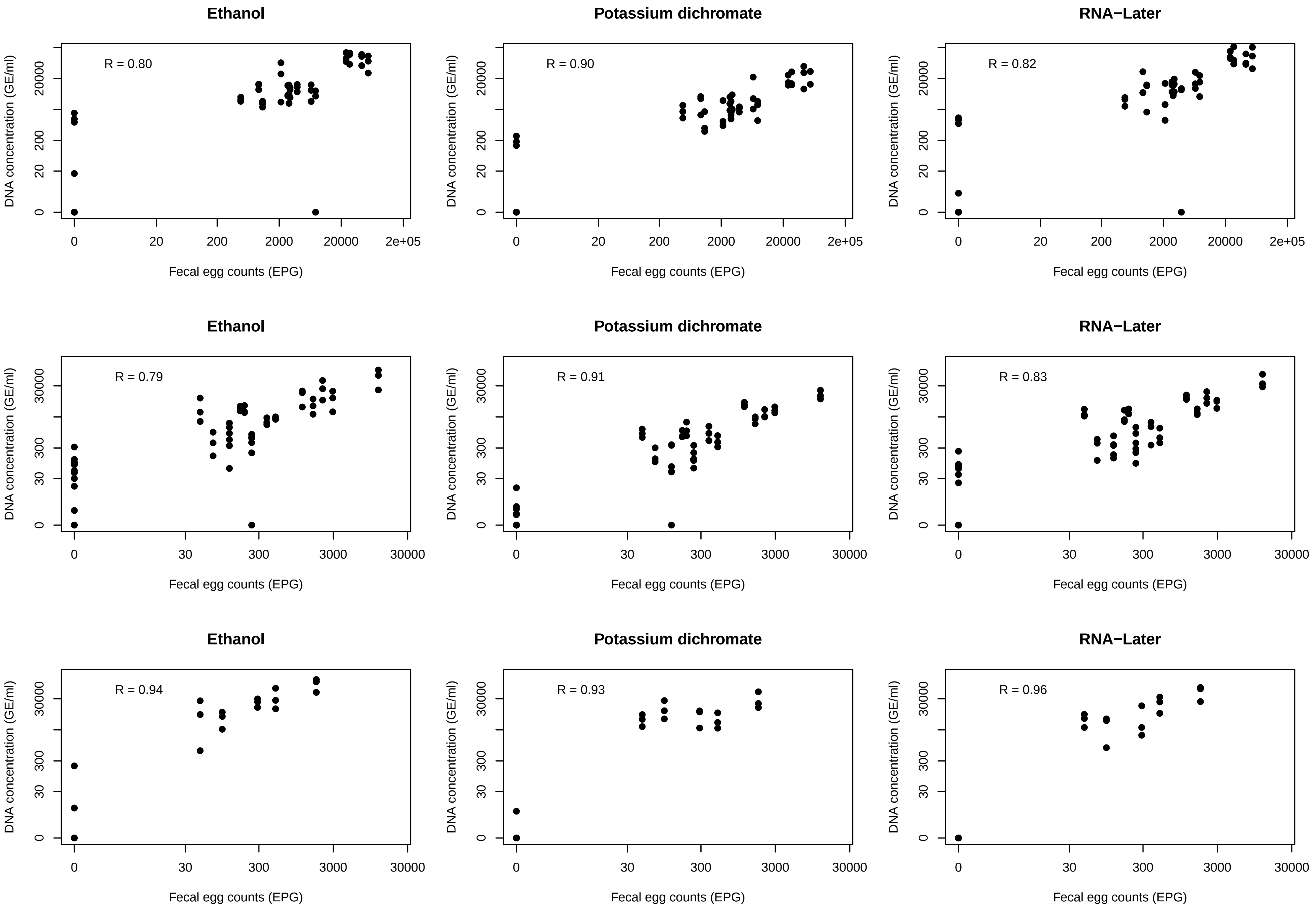
Ethanol
R = 0.80
Potassium dichromate
R = 0.90
RNA−Later
R = 0.82
Ethanol
R = 0.79
Potassium dichromate
R = 0.91
RNA−Later
R = 0.83
Ethanol
R = 0.94
Potassium dichromate
R = 0.93
RNA−Later
R = 0.96
DNA concentration (GE/ml)
Fecal egg counts (EPG)
0
20
200
2000
20000
2e+05
0
30
300
3000
30000
